# MDM2 Overexpression Modulates the Angiogenesis-Related Gene Expression Profile of Prostate Cancer Cells

**DOI:** 10.3390/cells7050041

**Published:** 2018-05-10

**Authors:** Thiagarajan Venkatesan, Ali Alaseem, Aiyavu Chinnaiyan, Sivanesan Dhandayuthapani, Thanigaivelan Kanagasabai, Khalid Alhazzani, Priya Dondapati, Saad Alobid, Umamaheswari Natarajan, Ruben Schwartz, Appu Rathinavelu

**Affiliations:** 1 Rumbaugh-Goodwin Institute for Cancer Research, Nova Southeastern University, Ft. Lauderdale, FL 33314, USA; tvenkatesan@nova.edu (T.V.); aa1836@mynsu.nova.edu (A.A.); sd975@nova.edu (S.D.); thanigaibio@gmail.com (T.K.); kalhazzani@gmail.com (K.A.); pd554@mynsu.nova.edu (P.D.); sa1402@mynsu.nova.edu (S.A.); rubenschwartz@yahoo.com (R.S.); 2 College of Pharmacy, Health Professions Division, Nova Southeastern University, Ft. Lauderdale, FL 33314, USA; 3 College of Medicine, Al-Imam Mohammad Ibn Saud Islamic University, Riyadh 13317, Saudi Arabia; 4 Post Graduate and Research Department of Biochemistry, Rajah Serfoji Government College, Thanjavur, TN 613005, India; aiyavu@yahoo.com; 5 College of Pharmacy, King Saud University, Riyadh 12371, Saudi Arabia; 6 VRR Institute of Biomedical Sciences, Kattupakkam, Chennai, TN 600056, India; ubaruthrarini@gmail.com

**Keywords:** MDM2, THBS1, MMP9, Angiogenesis, LNCaP-MST, PCR array, Nutlin-3

## Abstract

The Murine Double Minute 2 (MDM2) amplification or overexpression has been found in many tumors with high metastatic and angiogenic ability. Our experiments were designed to explore the impact of MDM2 overexpression, specifically on the levels of angiogenesis-related genes, which can also play a major role in tumor propagation and increase its metastatic potential. In the present study, we have used the human angiogenesis RT^2^ profiler PCR array to compare the gene expression profile between LNCaP and LNCaP-MST (MDM2 transfected) prostate cancer cells, along with LNCaP-MST cells treated with Nutlin-3, an MDM2 specific inhibitor. As a result of the overexpression of *MDM2* gene in LNCaP-MST (10.3-fold), Thrombospondin 1 (THBS1), Tumor necrosis factor alpha (TNF-α) and Matrix metallopeptidase 9 (MMP9) were also found to be significantly up-regulated while genes such as Epiregulin (EREG), Tissue inhibitor of metalloproteinases 1 (TIMP1) were down-regulated. Also, we determined the total MMP activity and MMP9 expression in LNCaP, LNCaP-MST and SJSA-1 cells. Our results indicated that MDM2 level is positively correlated with MMP activity and MMP9 secretion. Our findings offer strong supporting evidence that MDM2 can impact growth and metastatic potential of cancer cells through tilting the balance towards pro-angiogenic mechanisms.

## 1. Introduction

Most tumors contain either mutations or defects in the p53 pathway, which is considered as a major contributor to tumor development. An alternative mechanism is the overexpression of the *MDM2* gene, which can lead to the deactivation of p53 and its cell cycle control properties [1]. Interestingly, MDM2 level is up-regulated in nearly one-third of prostate cancers [2]. In fact, MDM2 negatively controls the protective activities of p53 through three major processes: (i) inhibition of the p53 transcriptional domain through direct binding, (ii) MDM2-facilitated proteasomal degradation of p53 via ubiquitination and, (iii) translocating p53 out of the nucleus to terminate its cell cycle control mechanisms. Hence, MDM2 oncogenic activities have been recognized as an essential element in promoting cancer progression. The MDMX protein, which is a homolog of MDM2, has also been identified as an inhibitor of p53 [3,4]. Although MDMX lacks the E3 ubiquitin ligase function, it can form a heterodimeric complex through its RING finger domain to inhibit p53 functions [5,6]. In the past few years, a group of imidazoline derivatives were developed to antagonize the MDM2-p53 interaction by binding to the N-terminal domain of MDM2 and reversing MDM2-mediated negative regulation of p53. In this context, Nutlin-3 has been studied extensively in the last fifteen years by utilizing various in vitro and in vivo models [7,8,9]. Several compounds which belong to this pharmacological family have also undergone clinical and pre-clinical investigations [10]. Indeed, accumulating evidence suggests that MDM2 can function through pathways that are independent of p53 and promote various tumorigenic activities. Therefore, antagonizing MDM2 protein represents an attractive approach to combat tumor growth and expansion.

Our previous studies have shown that MDM2 up-regulation triggers tumor angiogenesis [11] and thereby increases the metastatic ability through modulation of various pro-angiogenic pathways [12]. Multiple lines of evidence from various groups have proven vascular endothelial growth factor (VEGF) as the primary regulator of angiogenesis. Furthermore, our studies have suggested a strong correlation between MDM2 and VEGF in eight different cancer types, implicating a strong role for MDM2 in the regulation of angiogenesis process [12]. Also, our previous studies have clearly demonstrated that blocking MDM2 expression can inhibit the tube formation in the matrigel assay, that was performed with HUVEC cells, using the media obtained from the antisense oligo-treated GI-101A and HL-60 cells. In addition, MDM2 inhibition by AS5 antisense oligos caused a significant decrease in mRNA and protein levels of VEGF in both p53 mutant and p53 null cells (GI-101A and HL-60). These results further suggested a p53 independent role for MDM2 in regulating angiogenesis through VEGF-dependent pathways [13]. In this respect, multiple groups, have investigated the correlation between MDM2 and MMPs, which are known to influence VEGF pathways. However, many of the previously reported studies were done utilizing both in vitro and in vivo models, including pancreatic, liver, breast, and prostate cancer samples [14,15,16,17,18]. So far, none of the studies have assessed the role of MDM2 inhibition on MMPs expression nor its activities. Since the roles of MDM2 and its analog MDMX in cancer metastasis continue to evolve, we have conducted several studies on the levels of differentially expressed genes using LNCaP and LNCaP-MST prostate cancer cells. The LNCaP-MST cells are MDM2 transfected cells and therefore, they overexpress MDM2 at a higher level than LNCaP cells [12]. We suspected that MDM2 overexpression may impact angiogenesis via both p53 dependent and independent mechanisms. Therefore, we analyzed the expression levels of key genes in the angiogenic pathway using focused human angiogenesis RT^2^ profiler PCR array. Our experiments further support the hypothesis that MDM2 triggers angiogenesis mechanisms in LNCaP-MST cells. So far, all the existing literature and mounting evidence convincingly demonstrate a central role for MDM2 in promoting metastatic ability and tumorigenic potential. In this study, we present new data to confirm that angiogenesis mediators are differentially expressed in MDM2 transfected cells, which can be reversed by a pharmacological blocker as well as MDM2 silencing techniques.

## 2. Materials and Methods

### 2.1. Cell Culture and Nutlin-3 Treatment

LNCaP, LNCaP-MSI (MDM2 silenced cancer cells) and SJSA-1 (Osteosarcoma cells) were grown in RPMI-1640 medium and LNCaP-MST (MDM2 transfected cancer cells) were grown in Dulbecco’s Modified Eagle’s Medium (DMEM)-F12. The growth media for all cell lines were supplemented with 10% fetal bovine serum (FBS), 1% Amphotericin B and 1% Penicillin-Streptomycin. Cells were cultured in a humidified atmosphere with 95% air and 5% CO_2_. When LNCaP and LNCaP-MST cells reached 75–80% confluency, cells were treated with 20 µM Nutlin-3 for 24 h and used for RNA extraction to perform quantitative reverse transcription polymerase chain reaction (qRT-PCR) analysis.

### 2.2. Silencing of MDM2 in LNCaP-MSI Cells

Short hairpin RNA (shRNA) targeting human MDM2 and control vectors were obtained from Open Biosystems (Thermo Fisher Scientific, Inc., Waltham, MA, USA). LNCaP cells were selected with puromycin (8 μg/mL) for 15 days. The MDM2 silenced cells (LNCaP-MSI) were maintained in RPMI medium, according to standard protocols [19].

### 2.3. RNA Extraction

Total RNA was extracted from LNCaP, LNCaP-MST and Nutlin-3 treated LNCaP-MST cells. The RNA isolation was performed using the RNeasy mini-kit according to manufacturer’s protocol (Qiagen, Valencia, CA, USA). The purity and concentration of RNA were determined by measuring the ratio of absorbance at 260/280 nm.

### 2.4. Human Angiogenesis RT^2^ Profiler PCR Array

The cDNA of LNCaP, LNCaP-MST, and Nutlin-3 treated LNCaP-MST were synthesized using the RT^2^ first strand kit as per the company’s protocol, using the respective RNA as the templates (Qiagen, SABiosciences, Valencia, CA, USA). Gene expression profiling was conducted using the angiogenesis RT^2^ profiler PCR array (Catalog # PAHS-024 and PAHS-024Z, Qiagen, SABiosciences, Valencia, CA, USA). This array was designed to profile the expression of 84 key genes in the angiogenesis pathway. Quantitative reverse transcriptase PCR was conducted using the ABI StepOnePlus Real-time PCR Instrument (Applied Biosystems, Foster City, CA, USA) following the instructions of the array manufacturer. Relative gene expression was determined using the ∆∆C_T_ method. The heat map that was generated from the RT^2^ profiler data is showing the graphical representation of fold changes obtained by comparing the two groups. The heat map represents minimum and maximum gene expressions compared to the reference samples, where the intensity of the blocks is proportional to the degree of difference from the median.

### 2.5. Quantitative Reverse Transcription Polymerase Chain Reaction

Differences in the gene expression of selected genes such as THBS1, TNF-α, MDM2, CXCL10 (Chemokine (C-X-C motif) ligand 10), MMP9, EREG, TIMP1 and CXCL3 were compared between the LNCaP and LNCaP-MST cells. In addition, the RNA from Nutlin-3 treated LNCaP-MST cells were analyzed for THBS1, EREG and CXCL3 gene expressions. Calculation of Ct (threshold cycle) values for the amplification curves were achieved after subtracting β-actin values for normalization. Each qRT-PCR reaction volume of 20 μL consisted of 50 ng mRNA, 0.4 μM forward and reverse primers, 10 μL 2x SensiFAST SYBR Hi-ROX One-Step Mix, 0.2 μL Reverse transcriptase and 0.4 μL Ribosafe RNase inhibitors (10 U/µL) (Bioline, Taunton, MA, USA). The primer sequences used for qRT-PCR are listed in Table 1. The reaction was started with reverse transcription step at 45 °C for 10 min, denaturation at 95 °C for 2 min, and then the cDNA was amplified for 40 cycles with the denaturation at 95 °C for 5 s, annealing at 60 °C for 10 s and extension at 72 °C for 5 s. Each sample was amplified in triplicates on the ABI StepOnePlus Real-time PCR instrument (Applied Biosystems, Foster City, CA, USA).

### 2.6. Reverse Transcription Polymerase Chain Reaction (RT-PCR) Analysis of Gene Expression

For the purpose of confirming the changes related to the genes that were significantly altered, the RNA was extracted and then it was reverse transcribed using one-step reaction with the access RT-PCR system as described in our previous publication [12]. Amplification of specific genes such as THBS1, TNF-α, MMP9, CXCL10, EREG, TIMP1, MDM2, CXCL3 and β-actin were achieved using specific forward and reverse primers as listed in Table 1. The RT-PCR reaction products were separated on 1.5% agarose gel containing non-mutagenic fluorescent DNA dye (VWR Life sciences, Radnor, PA, USA) and the cDNA bands were visualized under UV light. The images were then captured and scanned using a Bioimaging system (UVP, Upland, CA, USA). The quantitative comparison of each band was achieved by measuring band intensity using the ImageJ program (NIH Image, Bethesda, MD, USA).

### 2.7. Protein Preparation and Western Blot Analysis

Cells were cultured in T-75 flasks until they reached 80% confluency. LNCaP, LNCaP-MST, LNCaP-MSI, and Nutlin-3 treated LNCaP-MST cells were lysed with RIPA (Radio-Immunoprecipitation Assay) lysis buffer containing protease inhibitor cocktail and sodium orthovanadate (Santa Cruz Inc., Dallas, TX, USA). Cell lysates were clarified by centrifugation at 4 °C for 20 min at 14,000 rpm and protein concentrations were quantitated using bicinchoninic acid (BCA) protein assay method (Thermo Fisher Scientific, Grand Island, NY, USA). For the western blot analysis equal concentration of protein was separated using sodium dodecyl sulfate-polyacrylamide gel electrophoresis (SDS-PAGE) and blotted onto a nitrocellulose membrane (GE Healthcare, Pittsburgh, PA, USA). Membranes were blocked using proteins from non-fat dry milk and probed with specific antibodies for MDM2 (Santa Cruz Inc., Dallas, TX, USA), TIMP1, MMP9, AKT, pAKT (Cell Signaling Technologies, Danvers, MA, USA), and β-actin (Sigma-Aldrich, Saint Louis, MO, USA). Finally, for the detection of specific proteins, the membranes were incubated in a solution containing LumiGLO Reserve Chemiluminescent substrate (KPL, Gaithersburg, MA, USA). Densitometric analyses were performed using the ImageJ program.

### 2.8. Cell Migration Assay

The effect of MDM2 inhibition on cell migration was assessed using scratch assay. For the migration assay, 12-mm transwell plates with 4 µm polycarbonate membrane inserts (Corning, NY, USA) were used. While performing the scratch assay, Human Umbilical Vein Endothelial Cells (HUVEC) were seeded on 6-well plates to 90–100% confluency. The cells were divided into three different groups: HUVECs, HUVECs co-cultured with LNCaP, or LNCaP-MST. All groups were cultured in the endothelial growth medium-2 (EGM-2). Subsequently, a sterile 200 µL tip was utilized to make a straight scratch line cell-free gap (~1-mm width) in each well for all groups (HUVECs, HUVECs co-cultured LNCaP and LNCaP-MST) and then they were treated with Nutlin-3 (10 µM). One subset from each group of cells was cultured in EGM-2 (without treatment) as a control. The images were captured to record the status of cells near the scratch lines at 0 h, 9 and 12 h post-scratch time points using Leica microscope (DMI 3000 B; Buffalo Grove, IL, USA).

### 2.9. MMP Activity Assay

The activity of MMPs were assessed in the serum-deprived supernatants of LNCaP, LNCaP-MST and SJSA-1 cells after Nutlin-3 (20 µM) treatment for 18 h. The MMP activity assay kit (Catalog # ab112146, Abcam, Cambridge, MA, USA) was used to analyze the activity of MMPs from cell supernatants by incubating with a fluorescent substrate [fluorescence resonance energy transfer (FRET) peptide]. The fluorescence was measured using a Synergy H1 hybrid microplate reader to calculate the enzymatic activity of MMPs (Biotek, Winooski, VT, USA).

### 2.10. Enzyme-Linked Immunosorbent Assay (ELISA) for MMP9

The expression levels of MMP9 was assessed in the serum-deprived supernatants of the LNCaP, LNCaP-MST and SJSA-1 cells after Nutlin-3 (20 µM) treatment using the ELISA method. The levels of MMP9 in cell supernatants were measured and quantified using human MMP9 Quantikine Immunoassay kit (R&D Systems, Minneapolis, MN, USA). All measurements were performed according to manufacturer’s protocol.

### 2.11. Statistical Analysis

The data presented are the mean ± SD and the statistical significance between the groups were analyzed by one-way analysis of variance (ANOVA) followed by Tukey’s multiple comparisons test. The *p*-value of less than 0.05 was considered as a statistically significant.

## 3. Results

### 3.1. Identification of Differentially Expressed Genes in LNCaP-MST Using Human Angiogenesis RT^2^ Profiler PCR Array

**Figure 1:**
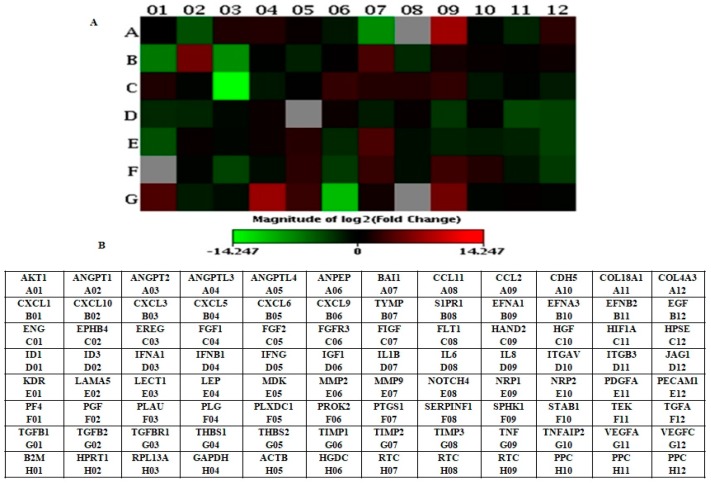
(**A**) Heat map showing differentially expressed genes in LNCaP-MST cells. The blocks represent high and low-level expressions respectively. The genes such as THBS1 and MMP9 (G-04 and E-07) were significantly up-regulated in LNCaP-MST cells. (**B**) Human angiogenesis gene table (PAHS-024) used in RT^2^ profiler PCR array experiments.

**Figure 2:**
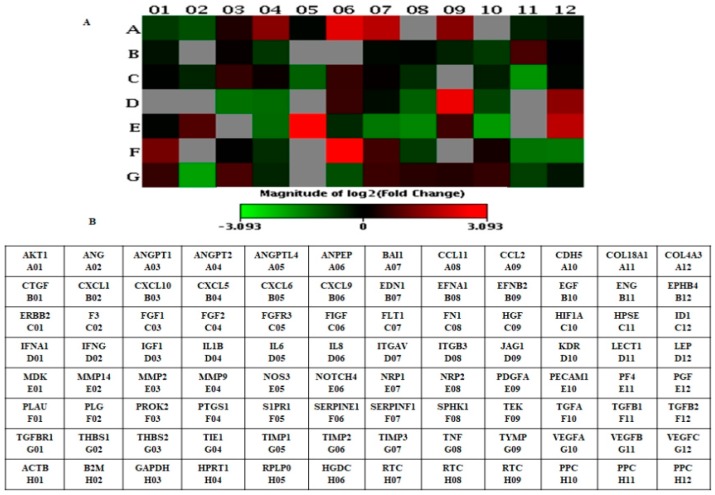
(**A**) Heat map showing differentially expressed genes in LNCaP-MST cells after Nutlin-3 treatment. (**B**) Human angiogenesis gene table (PAHS-024Z) used in RT^2^ profiler PCR array experiments.

RT^2^ profiler PCR array results confirm that genes were differentially expressed <1-fold (down-regulation) and >1-fold (up-regulation) in LNCaP-MST compared to the control LNCaP cells (Table 2). Nutlin-3 (20 µM) treatment reversed the THBS1 expression in LNCaP-MST cells (Table 3). The presented heat map clearly shows genes that were significantly altered in LNCaP-MST cells compared to LNCaP (control) and Nutlin-3 treatment of LNCaP-MST cells compared to untreated LNCaP-MST (Figures 1A,B and 2A,B). It is evident from these results that MDM2 overexpression can significantly influence the gene expression in LNCaP-MST cells compared to LNCaP. The THBS1 levels reversed by MDM2 specific inhibitor Nutlin-3 further confirmed these results in the subsequent qRT-PCR experiments.

### 3.2. Gene Expression in LNCaP-MST Using qRT-PCR

The qRT-PCR results substantiated the high expression levels of *MDM2* gene in LNCaP-MST cells compared to LNCaP. In addition, THBS1, TNF-α, CXCL10 and MMP9 were confirmed to be up-regulated and EREG, TIMP1 and CXCL3 were found to be most significantly down-regulated in LNCaP-MST compared to the LNCaP control. Furthermore, Nutlin-3 treatment impacted the gene expression in LNCaP-MST cells compared to untreated LNCaP-MST. In particular, THBS1 was down-regulated and EREG and CXCL3 were up-regulated, significantly after the inhibition of MDM2 using Nutlin-3 in LNCaP-MST cells (Table 4 and Table 5). Comparison of the mean Ct values between the two groups (LNCaP vs. LNCaP-MST; LNCaP-MST vs. LNCaP-MST treated with Nutlin-3) indicated significant up-regulation and/or down-regulation of the above-indicated genes, in support of our strong speculation that MDM2 might be responsible for the observed changes (Table 4 and Table 5). The results of the focused qRT-PCR changes paralleled the changes observed in the qPCR array analysis, reiterating the notion that differential gene expressions observed in above-mentioned cells and conditions are cancer growth related and are directly impacted by MDM2 status and functional ability (Table 2 and Table 3).

### 3.3. Determination of Gene Expression in LNCaP-MST Using RT-PCR

**Figure 3:**
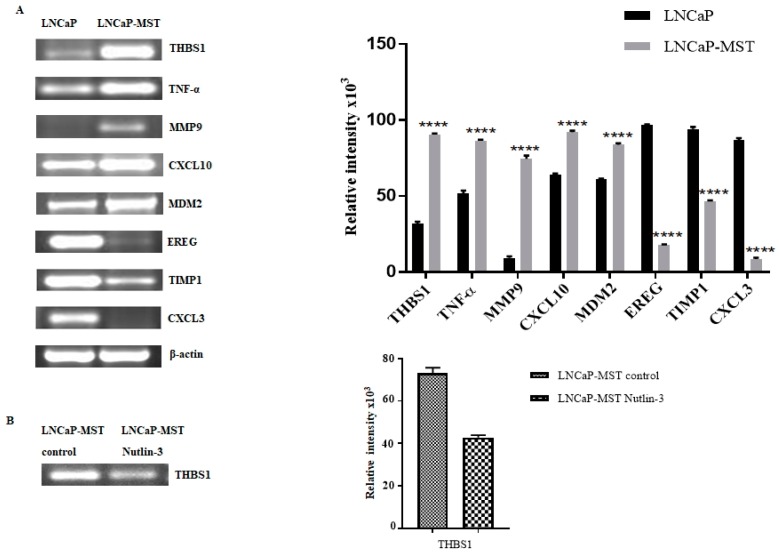
(**A**) RT-PCR pictures showing THBS1, TNF-α, MMP9, CXCL10, MDM2, EREG, TIMP1, CXCL3 and β-actin mRNA expression levels in LNCaP and MDM2 transfected prostate cancer cells and bar graph depicting the relative intensity of bands corresponding to differentially expressed genes in LNCaP and LNCaP-MST cells analyzed using ImageJ program. (**B**) Shows down-regulation of THBS1 after Nutlin-3 treatment. The densitometry analysis from at least three experiments are presented as mean ± SD and the levels of statistical significance are mentioned in comparison to respective controls (**** *p* < 0.0001).

To further confirm the results obtained from both PCR array and qRT-PCR experiments, the RT-PCR was also carried out using specific primers. In the way of reinforcing our results, Figure 3A shows the expression levels of selected angiogenesis-related genes in LNCaP and LNCaP-MST and the corresponding bands of the genes with relative intensities using densitometric analysis in up-regulated or down-regulated conditions. Because of the differences in the RNA levels, the difference in band intensities obtained through RT-PCR for up-regulated genes were THBS1, TNF-α, MMP9, CXCL10 and MDM2 respectively in LNCaP-MST cells compared to LNCaP cells. In the down-regulated category, a decrease in band intensities was detected in EREG, TIMP1 and CXCL3 respectively in MDM2 transfected LNCaP-MST cells when compared to the LNCaP cells. On the other hand, in Nutlin-3 treated LNCaP-MST cells, THBS1 mRNA expression was reduced significantly (Figure 3B) suggesting that inhibition of MDM2 by Nutlin-3 is the direct cause of THBS1 suppression in LNCaP-MST cells.

### 3.4. Levels of Protein Expression in LNCaP, LNCaP-MST, LNCaP-MSI and Expression Modulated by Nutlin-3 Treatment

**Figure 4:**
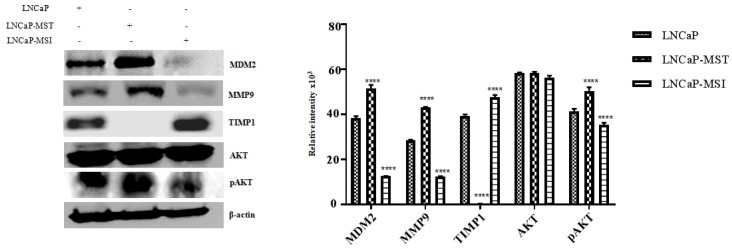
Levels of MDM2, MMP9, TIMP1, AKT and pAKT protein expression in LNCaP and LNCaP-MST and LNCaP-MSI cells. In these western blotting experiments, the expression level of β-actin was used as a loading control (**** *p* < 0.0001).

**Figure 5:**
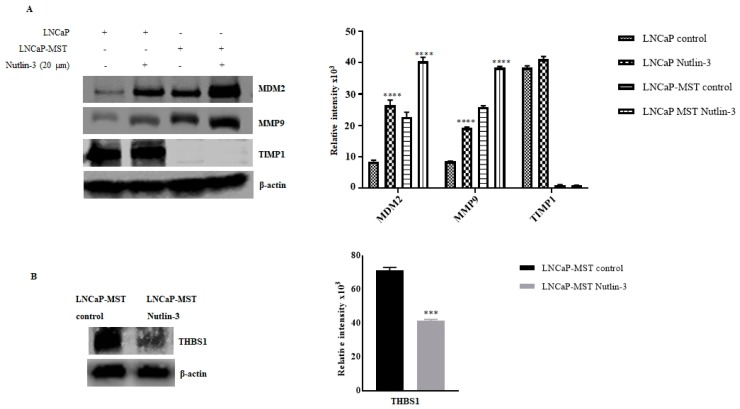
(**A**) Illustrates that the MDM2, MMP9 and TIMP1 protein expression in the LNCaP and LNCaP-MST cells after Nutlin-3 (20 µM) treatment (**** *p* < 0.0001). (**B**) Shows down-regulation of THBS1 protein after Nutlin-3 treatment (*** *p* < 0.001).

In addition to analyzing the gene expression levels through determining the RNA status, the levels of the corresponding protein were also analyzed starting with MDM2. In addition to the increase in the level of MDM2, caused by transfection, the levels of MMP9 and pAKT were also higher in LNCaP-MST compared to LNCaP control. Coincidentally, the levels of these proteins were found to be significantly down-regulated in MDM2 silenced LNCaP-MSI cells (Figure 4). Interestingly, the TIMP1 protein levels were significantly down-regulated in LNCaP-MST cells with no treatment or changes in the growth conditions, which may contribute to some of the pro-angiogenic, and pro-metastatic mechanisms that are known to be activated by MDM2 overexpression. In addition, we were not able to find any change in the total expression of AKT (Figure 4) but only in the pAKT levels implying the possibility that any changes the impact in this pathway by MDM2 must be due to altered phosphorylation in addition to the PTEN loss that is found in LNCaP cells. In further support of our hypothesis that MDM2 is the primary cause for the observed changes in expressions of selected key genes, an increased level of TIMP1 expression was found in the MDM2 silenced cells (Figure 4). In fact, Nutlin-3 treatment significantly increased the expression of MDM2 and MMP9 in LNCaP and LNCaP-MST cells. However, Nutlin-3 treatment did not reverse TIMP1 expression in the above listed cells (Figure 5A). On the other hand, similar to the transcriptional inhibition of THBS1, the protein expression of THBS1 was significantly down-regulated after Nutlin-3 treatment (Figure 5B).

### 3.5. Role of MDM2 on HUVECs Migration and Anti-Migratory Effects of Nutlin-3 Treatment

**Figure 6:**
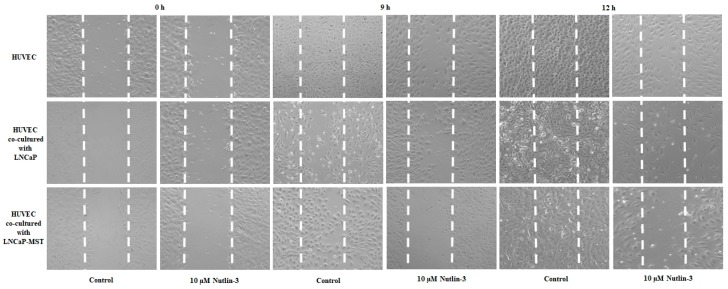
Effects of co-culturing MDM2 expressing cells with HUVECs on the migration of HUVECs before and after Nutlin-3 (10 µM) treatment was assessed. Scratch assay was carried out to assess cell migration ability of HUVECs. After making the scratch lines, images were captured at 0, 9 and 12 h.

To assess whether MDM2 expressing cells influence the migration ability of HUVECs, a scratch assay in HUVECs co-cultured with LNCaP or LNCaP-MST was performed before and after Nutlin-3 treatment. After 9 and 12-h treatments, LNCaP-MST showed an augmented migratory impact on HUVECs compared to LNCaP. The Nutlin-3 treatment was able to significantly reduce the migration of HUVECs co-cultured with LNCaP compared to HUVECs control (Figure 6).

### 3.6. Impact of MDM2 and Nutlin-3 Treatment on MMP Activity

**Figure 7:**
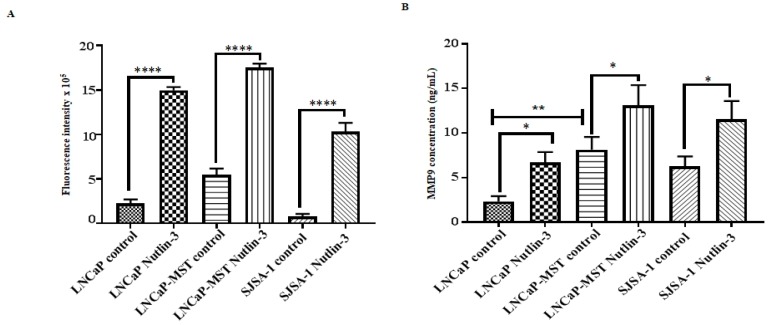
(**A**) The role of MDM2 expressing cells in MMP activity in LNCaP, LNCaP-MST, SJSA-1 and after Nutlin-3 (20 µM) treatment. Results showed that MMPs fluorescence intensity corresponded to the MMPs activity from cell culture supernatant (**** *p* < 0.0001). (**B**) MMP9 ELISA from cell culture supernatant displayed a significant increase in MMP9 secretion in MDM2 transfected LNCaP-MST compared to LNCaP and Nutlin-3, which increased the secretion of MMP9 in LNCaP, LNCaP-MST, and SJSA-1 (* *p* < 0.05) (** *p* < 0.01).

To determine the MMP activity in the supernatant of MDM2 expressing cells, conditioned medium was collected from cells treated with and without Nutlin-3 and incubated with MMP fluorescence substrate. As shown in Figure 7A, the fluorescence intensity corresponding to MMP activity is significantly increased in LNCaP-MST compared to LNCaP. On the other hand, treatment with Nutlin-3 showed an increase in the fluorescence intensity in all MDM2 harboring cells including LNCaP, LNCaP-MST and SJSA-1.

### 3.7. Effect of MDM2 and Nutlin-3 Treatment on MMP9 Expression

The secreted level of MMP9 in MDM2 expressing cells was determined from the conditioned medium that was collected from cells treated with and without Nutlin-3. As shown in Figure 7B, MMP9 secretion was significantly increased in MDM2 transfected cells (LNCaP-MST) compared to the non-transfected LNCaP cells. However, Nutlin-3 did not reverse the secretion of MMP9 but instead increased the level of MMP9 secretion in all MDM2 overexpressing cells.

## 4. Discussion

LNCaP cells utilized in this study have been used as a subject of analysis for their tumor potency and various other characteristics, including pro-angiogenic abilities. Similarly, LNCaP-MST cells have been extensively studied for their greater angiogenic potential in various laboratories including our own laboratory. Recently, we have demonstrated with sufficient evidence that some of the key genes such as HIF-1α and p300, which are essential for angiogenesis, are also overexpressed in MDM2 transfected LNCaP-MST cells compared to non-transfected LNCaP cells [12]. These findings that were reported earlier as well as our current findings are consistent with our long-standing speculation that MDM2 overexpression is manifested through the impact on many different pathways in highly metastatic and recurrent cancers [20]. Therefore, our gene expression profiling experiments were designed to understand the impact of MDM2 overexpression on the levels of multiple genes impacting angiogenesis mechanisms, such as THBS1, TNF-α, CXCL10 and MMP9, which have a major role in pathways related to the regulation of growth rate and metastasis. An important promoter of the metastatic process is the Matrix Metallopeptidases (MMPs) [21], which are reported to be overexpressed in many solid tumors [22]. In addition, the confirmation that MMP9 is involved in triggering the angiogenic switch came from a multistage pancreatic carcinogenesis model of transgenic RIP1-Tag2 mice in which both MMP2 and MMP9 were found to be up-regulated, rendering good correlation with the metastatic ability of a wide range of cancer cells [23]. The increase in the level of MMP9 observed in the LNCaP-MST cells in our study might also be due to the increased expression of THBS1, TNF-α and CXCL10 which correlates positively with the increase in p300/CBP and STAT3 observed while there was a concomitant decrease of TIMP1 levels. Though TSP1 (aka THBS1) is recognized as an anti-angiogenic factor, it has also been shown to promote angiogenesis through the stimulation of endothelial cell migration [24]. In this context, the increase in THBS1 expression has been found to be associated with the enhancement of angiogenic properties in the gastric carcinoma model [25] and more importantly, it has been reported to up-regulate the expression of MMP9 in breast cancer cells [26]. Therefore, we further emphasized our speculation that the concomitant up-regulation of MMP9 and THBS1 found in the LNCaP-MST cells may be due to MDM2 overexpression that can easily lead to the acquisition of aggressive tumor phenotype with increased metastatic potential [27]. This conclusion is additionally evidenced by the MDM2 inhibition results obtained using Nutlin-3 and the gene silencing approach that was utilized in the LNCaP-MSI cells that forced decreases in THBS1 and MMP9 expressions respectively. In regulating MMP9, the TIMPs are known to have important roles, in particular, both TIMP1 and TIMP2 (Tissue inhibitor metallopeptidase 2) have the ability to inhibit the enzyme activity of MMP9 and MMP2 [28,29] by binding in the proximity of the catalytic domain of MMPs and interfering with the substrate attachment to their catalytic site [30]. A significant down-regulation of TIMP1 that was observed in LNCaP-MST cells correlates well with the up-regulation of MMP9 through an inverse intracellular mechanism similar to the finding reported by several other laboratories. Though Nutlin-3 treatment did not alter the TIMP1 gene expression levels in LNCaP-MST cells, the increase in TIMP1 protein expression observed in MDM2 silenced LNCaP-MSI cells offer sufficient support to our speculation related to MDM2 ability to impact TIMP1 and MMP9 levels.

Another gene that was found to be significantly elevated in LNCaP-MST cells was *TNF-α*. This cytokine is produced as an endogenous tumor promoter to trigger a wide range of pre-cancerous effects in various tumors [31]. The role of TNF-α in promoting several tumorigenic activities, including invasiveness, angiogenesis, and metastasis of many cancers has been recognized [32]. Our results clearly show an up-regulation in MDM2-transfected LNCaP-MST cells, which in turn, can favor increased angiogenesis triggered by MDM2 through an additional pathway. Interestingly, the CXCL10, which has been reported to up-regulate MMP9, requires additional studies using our experimental model because some of the previous reports have indicated that both chemokines CXCL4 [chemokine (C-X-C motif) ligand 4] and CXCL10 can act as inhibitors of angiogenesis [33]. This would make the correlation between the expression of above-mentioned chemokines and the process of tumor angiogenesis as inverse; however, our LNCaP-MST cells show an opposite correlation. Similarly, the pro-angiogenic cytokines such as CXCL1 [chemokine (C-X-C motif) ligand 1], CXCL3, and CXCL6, [chemokine (C-X-C motif) ligand 6], which are typically expressed in highly aggressive cancer, were found to be down-regulated in LNCaP-MST cells. Therefore, it is not clear whether CXC chemokines have less impact on the growth ability or are controlled by a feedback mechanism triggered by MDM2 leading to levels showing an inverse correlation. Contrary to our expectation, Nutlin-3 did not reduce the expression of MMP9 or the activity of MMPs in our in vitro models but instead increased the secretion of MMP9 and the total MMPs activity. The increased activity and expression of the MMPs in our study can be attributed to many factors including p53 dependent or independent mechanisms. It is important to note that blocking of p53 binding site by Nutlin-3 leads to accumulation of MDM2 that occurs as a result of a feedback loop that is triggered by the transcriptional activation of p53 [34]. Though MDM2 level is elevated, it is suspected that the presence of Nutlin-3 in the system should neutralize its function. In addition, our results have suggested that MDM2 can positively correlate with TNF-α expression to modulate MMP9 levels. Similarly, a few other studies have also reported an increase in TNF-α and CXCL3 levels following Nutlin-3 treatment. In fact, TNF-α has been shown to positively induce MMP9 expression and secretion in various cancer cells to promote their metastatic ability [35,36]. Thus, our overall results substantiate the possibility that MDM2 can promote the expression of MMP9 through activation of TNF-α pathway to support the invasive behavior of the cancer cells. Our results have also revealed that HUVEC co-cultured with Nutlin-3 treated LNCaP-MST cells are less migratory compared to the non-treated cells. Thus, our results suggest that MDM2 inhibition can suppress the paracrine function of the cancer cells.

As evidence by the results of our three-pronged analysis, EREG, which is a member of the epidermal growth factor (EGF) family [37] is also impacted by MDM2 in the LNCaP-MST cells. A high-level expression of EREG mRNA has been reported in several human cancer cell lines, which has established EREG also as an important regulator of cancer growth and metastasis [38]. Increased expression or activity of EREG has been reported to contribute to the progression of many tumors, including colon and breast [39]. However, the decreased expression levels of EREG observed in LNCaP-MST cells does not seem to follow the typical pattern that was previously reported but certainly appears to be impacted by MDM2 expression. More interestingly, the treatment of cells with Nutlin-3 significantly reverses the expression of not only EREG, but also CXCL3 expressions in LNCaP-MST cells. One of the possibilities that cannot be ruled out at this time is, MDM2 may be negatively impacting the transcriptional mechanisms regulated by AP2-α (Activating enhancer binding protein 2 alpha) promoter complex, which has been shown to downregulate CXCL1, CXCL2 [chemokine (C-X-C motif) ligand 2], and EREG levels in HeLa cells [40,41]. Finding an explanation for some of the contradictions at the intracellular level requires additional studies.

## 5. Conclusions

Finally, based on our current findings and strong supporting evidence that can be derived from previous reports, it is quite evident that some of the discussed changes observed in LNCaP-MST cells through the RT^2^ gene expression Profiler are due to MDM2 overexpression, whereas Nutlin-3 treatment can reverse the mRNA levels of THBS1, EREG, and CXCL3. Our results offer additional confirmation to our speculation that MDM2 can shift the balance of the intracellular events in cancer cells towards pro-angiogenic mechanisms through up-regulation of TNF-α, MMP9, and CXCL10. Adding to the existing body of evidence, our data offer further support to the notion that MDM2 can produce several p53-independent mechanisms and, therefore, is emerging as a significant therapeutic target than ever before. Additionally, some of the differentially expressed genes in MDM2 overexpressing cancers can be utilized as a significant biomarker in conjunction with their master regulator. Therefore, identifying the exact mechanisms by which MDM2 is impacting cancer progression at the molecular level would offer greater benefits towards treating cancers that are overexpressing MDM2.

## Figures and Tables

**Table 1:** Primer sequences.

Transcript	Forward and Reverse Primers
**qRT-PCR Primers**	
THBS1	Forward: 5′-AGCGTCTTCACCAGAGACCT-3′
	Reverse: 5′-CATTCACCACGTTGTTGTCA-3′
TIMP1	Forward: 5′-TACTTCCACAGGTCCCACAA-3′
	Reverse: 5′-ATTCCTCACAGCCAACAGTG-3′
CXCL3	Forward: 5′-CCACACTCAAGAATGGGAAG-3′
	Reverse: 5′-CTGTCCCTAGAAAGCTGCTG-3′
MDM2	Forward: 5′-CACCTCACAGATTCCAGCTT-3′
	Reverse: 5′-CGCCAAACAAATCTCCTAGA-3′
β-actin	Forward: 5′-GGACTTCGAGCAAGAGATGG-3′
	Reverse: 5′-AGCACTGTGTTGGCGTACAG-3′
**RT-PCR Primers**	
THBS1	Forward: 5′-GACTAGGCGTCCTGTTCCTG-3′
	Reverse: 5′-ACCTGGCCAGAGTGGTCTTT-3′
TIMP1	Forward: 5′-GGACACCAGAAGTCAACCAGACC-3′
	Reverse: 5′-CGTCCACAAGCAATGAGTGCC-3′
CXCL3	Forward: 5′-GCAGGAGCGTCCGTGGTCAC-3′
	Reverse: 5′-GCTCTGGTAAGGGCAGGGACC-3′
MDM2	Forward: 5′-CTGGGGAGTCTTGAGGGACC-3′
	Reverse: 5′-CAGGTTGTCTAAATTCCTAG-3′
β-actin	Forward: 5′-GTGGGGCGCCCCAGGCACCA-3′
	Reverse: 5′-CTCCTTAATGTCACGCACGATTTC-3′
**Primers Used for Both qRT-PCR and RT-PCR**	
TNF-α	Forward: 5′-TCCTTCAGACACCCTCAACC-3′
	Reverse: 5′-AGGCCCCAGTTTGAATTCTT-3′
MMP9	Forward: 5′-CTCTGGAGGTTCGACGTG-3′
	Reverse: 5′-GTCCACCTGGTTCAACTCAC-3′
CXCL10	Forward: 5′-GCTTAGACATATTCTGAGCCTAC-3′
	Reverse: 5′-AGCTGATTTGGTGACCATCATTG-3′
EREG	Forward: 5′- TCCATCTTCTACAGGCAGTCC-3′
	Reverse: 5′-CACGGTCAAAGCCACATACTC-3′

**Table 2:** List of selected differentially expressed genes modulated by MDM2 transfection in LNCaP prostate cancer cells, analyzed using PCR array.

Gene	Accession Number	Description	Fold Change
**Up-Regulated Genes**			
THBS1	NM_003246	Thrombospondin 1	155.3
CXCL10	NM_001565	Chemokine (C-X-C motif) ligand 10	41.5
TNF	NM_000594	Tumor necrosis factor	40.6
MMP9	NM_004994	Matrix metallopeptidase 9 (gelatinase B, 92 kDa gelatinase, 92 kDa type IV collagenase)	11.5
**Down-Regulated Genes**			
EREG	NM_001432	Epiregulin	0.0001
TIMP1	NM_003254	TIMP metallopeptidase inhibitor 1	0.0007
CXCL3	NM_002090	Chemokine (C-X-C motif) ligand 3	0.004
CXCL1	NM_001511	Chemokine (C-X-C motif) ligand 1 (melanoma growth stimulating activity, alpha)	0.009
CXCL6	NM_002993	Chemokine (C-X-C motif) ligand 6 (granulocyte chemotactic protein 2)	0.24

**Table 3:** Analysis of gene expression in LNCaP-MST cells after Nutlin-3 treatment using PCR array.

Gene	Accession Number	Description	Fold Change
**Down-Regulated Gene**			
THBS1	NM_003246	Thrombospondin 1	0.26

**Table 4:** Analysis of qRT-PCR for selected genes that are up-regulated and down-regulated in LNCaP-MST cells compared to the LNCaP cells.

Gene	ΔCt: LNCaP	ΔCt: LNCaP-MST	Fold Change
**Up-Regulated Genes**			
THBS1	12.9	5.9	125.8
TNF-α	10.2	6.4	14.3
MDM2	5.1	1.8	10.3
CXCL10	22.6	20.2	5.2
MMP9	12	11.5	1.4
**Down-Regulated Genes**			
EREG	3.5	13.4	0.001
TIMP1	2.9	10.3	0.006
CXCL3	5.8	13.4	0.005

**Table 5:** Analysis of qRT-PCR in LNCaP-MST cells after Nutlin-3 treatment.

Gene	ΔCt: LNCaP-MST	ΔCt: LNCaP-MST	Fold Change
	Control	Nutlin-3 Treated	
**Up-Regulated Genes**			
EREG	16.7	15.2	2.8
CXCL3	15.9	14.2	3.4
**Down-Regulated Genes**			
THBS1	**5**	7.4	0.18
